# In retinitis pigmentosa TrkC.T1-dependent vectorial Erk activity upregulates glial TNF-α, causing selective neuronal death

**DOI:** 10.1038/s41419-017-0074-8

**Published:** 2017-12-14

**Authors:** Alba Galán, Sean Jmaeff, Pablo F. Barcelona, Fouad Brahimi, Marinko V. Sarunic, H. Uri Saragovi

**Affiliations:** 1 Lady Davis Institute-Jewish General Hospital, McGill University, Montréal, QC H3T 1E2 Canada; 2 Department of Pharmacology and Therapeutics, McGill University, Montréal, QC Canada; 3 School of Engineering Science, Simon Fraser University, Burnaby, BC Canada; 4 Department of Ophthalmology, McGill University, Montréal, QC Canada

## Abstract

In some diseases the TrkC.T1 isoform is upregulated in glia, associated with glial TNF-α production and neuronal death. What remains unknown are the activating signals in glia, and how paracrine signals may be selective for a targeted neuron while sparing other proximate neurons. We studied these questions in the retina, where Müller glia contacts photoreceptors on one side and retinal ganglion cells on the other. In a mutant Rhodopsin mouse model of retinitis pigmentosa (RP) causing progressive photoreceptor death—but sparing retinal ganglion cells—TrkC.T1 and NT-3 ligand are upregulated in Müller glia. TrkC.T1 activity generates p-Erk, which causes increased TNF-α. These sequential events take place predominantly in Müller fibers contacting stressed photoreceptors, and culminate in selective death. Each event and photoreceptor death can be prevented by reduction of TrkC.T1 expression, by pharmacological antagonism of TrkC or by pharmacological inhibition Erk. Unmasking the sequence of non-cell autologous events and mechanisms causing selective neuronal death may help rationalize therapies.

## Introduction

Retinitis pigmentosa (RP) is an inherited degenerative retinal disease characterized by progressive apoptosis of photoreceptors that ultimately leads to irreversible loss of vision^1^. Photoreceptor death can be triggered by one of more than 250 gene mutations^2–4^. There are no effective treatments that can halt or reverse the disease due to the extremely heterogeneous nature of the mutations and the poor understanding of molecular mechanisms that cause photoreceptor cell death. Here, we investigate a disease mechanism that could yield a disease-modifying target.

Inflammatory events such as expression of glial tumor necrosis factor-α (TNF-α) are clearly involved in photoreceptor degeneration^5, 6^. The mechanisms that activate retinal TNF-α production in disease remain unclear, and may involve the mitogen-activated-kinase (MAPK) extracellular signal-regulated protein kinases 1 and 2 (ERK1/2)^7,8^. Phosphorylation/activation of ERK1/2 (p-Erk) can play multiple and seemingly opposite roles from pro-survival to pro-death/pro-inflammatory pathways^9^. However, very few studies have ascribed a clear function to ERK1/2 in the injured retina^10^ and the underlying mechanisms leading to ERK1/2 activation during disease remain unknown.

Neurotrophins are a family of protein growth factors—nerve growth factor (NGF), brain-derived neurotrophic factor (BDNF) and neurotrophin-3 (NT-3)—that regulate the nervous system and other tissues. All the neurotrophins bind the p75 receptor whose function is generally associated with pro-death signals in many neurodegenerative diseases, including retinopathies^11–15^. The neurotrophic activities are mediated by selective ligand binding to the Trk family of receptor tyrosine kinases (TrkA, TrkB, TrkC and p75). NGF binds to TrkA, BDNF binds preferentially to TrkB^16^, whereas NT-3 interacts mainly with TrkC but also with TrkA^17^. Survival is mediated by full-length kinase active Trk receptors^18–21^, but there are also truncated isoforms that lack kinase activity.

Here, we postulated that a truncated TrkC receptor isoform (TrkC.T1) may be implicated in RP pathology, in analogy to other neurodegenerative diseases^22,23^. A single *TrkC* locus transcribes mRNA encoding for full-length TrkC (TrkC-FL), and the mRNA can be alternatively spliced to a truncated isoform TrkC.T1 mRNA that lacks the kinase domain and gains a short new intracellular domain with new signaling properties. Both TrkC-FL and TrkC.T1 are bound and activated by the growth factor NT-3. However, ligand activation of each receptor yields a different functional outcome. Because TrkC.T1 is upregulated in disease^22,23^, its signals prevail. TrkC.T1 has a dominant negative function upon TrkC-FL^24,25^, and can activate Rac1 signals^26^ to cause neuronal death in a paracrine manner^22,23^.

For example, in glaucoma models caused by high intraocular pressure, TrkC.T1-dependent secretion of TNF-α by Müller cells is etiological to the degeneration of retinal ganglion cells (RGCs)^22^, the neuronal population specifically affected in this disease. It is intriguing that while RGCs die in a TrkC.T1-dependent manner, other neuronal populations remain unaffected in early stages of glaucoma. This is especially curious given that TrkC.T1 expression occurs in Müller glia and not in injured RGCs.

Here, we use the RHOP347S (Rhodopsin mutant, RHOP) mouse model of RP to examine the mechanism of TrkC.T1 in the *selective* death of photoreceptors, the neuronal population specifically affected in this disease, and to elucidate TrkC.T1 intracellular signaling events leading to TNF-α-mediated photoreceptor death. We provide genetic, anatomical and pharmacological evidence showing that during RP disease Müller glia upregulates TrkC.T1 and its ligand NT-3, to activate p-Erk, causing increased TNF-α, which induces photoreceptor degeneration. The mechanism accounting for the selective impact on photoreceptors may relate to these signals in Müller cell fibers being “vectorial” in the direction of stressed photoreceptors. Our data point to a non-cell autologous or paracrine mechanism of photoreceptor cell death. This is the first evidence that shows the relevance of TrkC.T1 in RP, and disease mechanisms that may help rationalize therapies for retinal degenerative pathologies.

## Materials and methods

### Cell lines

HEK293 cells were transfected with human full-length *TrkC* cDNA (293-TrkC-FL) or with rat *TrkC.T1* cDNA (293-TrkC.T1). The cells are stably transfected subclones that express high levels of TrkC-FL or TrkC.T1 receptors and are respectively grown under drug selection (0.5 mg/ml G418 or 1 mg/ml Puromycin). The rat glial cell line, rMC-1, was kindly donated by Dr. Adriana Di Polo. rMC-1 has been previously characterized^27^.

### RNA interference knockdown of TrkC.T1

A short hairpin RNA (shRNA) specifically targeting a unique 3′ sequence of the TrkC.T1 mRNA was designed using the DSIR algorithm (http://biodev.cea.fr/DSIR/DSIR.html). The TrkC.T1-targeting shRNA sequence 5′-GGACAATAGAGATCATCTAGT-3′, or a scrambled control sequence 5′-CCTAAGGTTAAGTCGCCCTCG-3′ were cloned into a pLKO.1 lentiviral shRNA-expression vector. pLKO.1^scrambled^ and pLKO.1^TrkC-T1^ lentiviral vectors were packaged in HEK-293T cells and active viral particles were purified. rMC-1 cells were then transduced with lentiviral particles, and selected with 1 mg/ml puromycin. TrkC.T1-specific depletion (and no effect upon TrkC-FL expression) was determined by real-time quantitative PCR and by western blotting. Levels of p-Erk and p-Akt were quantified in rMC-1 cells treated with NT-3, and to assess TrkC.T1 dependence its expression was silenced using PLKO.1TrkC.T1 lentivirus that destabilizes TrkC.T1 mRNA^23^.

### Animal models

All animal procedures respected the institutional animal care and use committee guidelines for use of animals in research, and to protocols approved by McGill University Animal Welfare Committees. All animals were housed under a 12 h dark–light cycle with food and water ad libitum. Wild-type (WT) healthy C57/BL6 mice were used as experimental controls. Previously, we used the truncated TrkC.T1 knockout mouse^22^, and the “RHOP347S” transgenic mouse (Rhodopsin mutant) model of retinitis pigmentosa (termed RHOP in the experimental designs) was kindly donated by Dr. T. Li. Rhodopsin mutant is exclusively expressed in photoreceptors and generates stress in these cells due to protein misfolding. Both mice models were consistently backcrossed onto a pure C57BL/6J (B6) background.

The RHOP347S (RHOP) mice were crossed with homozygous TrkC.T1 knockout mice (TrkC.T1^*−/−*^) to generate the RHOP:TrkC.T1^+/*−*^ genotype (retinitis pigmentosa-diseased mice, TrkC.T1 heterozygous; hereafter RHOP:T1). These mice express the RHOP mutant gene, lack the ability to produce TrkC.T1 mRNA from one allele and have 50% less TrkC.T1 protein. Homozygous TrkC.T1^*−*/*−*^ mice do not have any abnormality and are viable^22^. However, the complete deletion of TrkC.T1 (TrkC.T1^*−*/*−*^) in RHOP mice yields small size litters and low-weight offspring, with some mice showing abnormal small eyes and do not open the eye lids normally. Therefore, only heterozygous RHOP:T1 mice were used experimentally.

### Genotypic screening

We used a PCR-based method for subsequent genotyping of the animals. Screening of TrkC.T1 was done using the same conditions as previously described in our laboratory^22^. We used the following primers: RM015 Rho forward (F) 5′-GGATTCTGTTTGACATGGGG-3′ and RM016 Rho reverse (R) 5′-TCCAGTCAGGACTCAAACCC-3′ for RHOP mice screening, and F 5′-ACCACAGTCCATGCCATCAC-3′ and R 5′-TCCACCACCCTGTTGCTGTA-3′ for GAPDH controls. PCR reactions were done using the “Extracta^TM^ DNA Prep for PCR-Tissue” kit (Quanta Biosciences, MD, USA). All PCR conditions were performed as follows: 35 cycles at 96 °C for 2 min, 94 °C for 30 min, 58 °C for 30 min; 35 cycles at 72 °C for 40 min and 72 °C for 7 min.

### Intravitreal injections

Intravitreal injections were performed as previously described^12^. Briefly, mice were anesthetized with 3% isoflurane, delivered through a gas anesthetic mask. The drugs were delivered using a Hamilton syringe. Injections were done using a surgical microscope to visualize the Hamilton entry into the vitreous chamber and confirm delivery of the injected solution. After the injection, the syringe was left in place for 30 s and slowly withdrawn from the eye to prevent reflux. Experimental right eyes were injected with the test agents and control left eyes serve as internal control.

### Drug regimen

#### Pharmacological inhibition

All intravitreal injections delivered 2 µl of the MAPK/ERK inhibitor PD98059 (10 mM stock) or the selective-TrkC antagonist KB1368 (1 mM stock). Control eyes were injected with 50% dimethyl sulfoxide (DMSO) vehicle for PD98059 assays or 5% DMSO vehicle for KB1368 assays. Intravitreal injections in WT or RHOP mice were done at postnatal day 17 (PN 17). Experimental time points to measure outer nuclear layer (ONL) thinning were set at PN days 18, 22, 24 and 28. The effect of the compounds on the expression of p-Erk and p-Akt was analyzed at PN day 18 (24 h after intravitreal injection).

#### NT-3 stimulation

Cells were uninfected (control) or infected with control lentivirus pLKO-1^scrambled^, or with pLKO-1^TrkC.T1^ that specifically reduces TrkC.T1 mRNA. Cells were serum-starved for 1 h, and then treated with vehicle or 4 nM NT-3 for 10 min. Then, cells were collected and processed for western blot analysis.

### Optical coherence tomography imaging

We used noninvasive spectrometer-based Fourier-domain optical coherence tomography (FD-OCT) techniques in longitudinal studies to quantify the structural changes occurring during the progressive retinal degeneration in the RHOP transgenic model of RP. FD-OCT is a noninvasive method that allows time-kinetic studies in the same animal, with axial resolution in tissue nominally better than 3 μm and repeatability of the measurements from B-scans better than 1 μm. Data acquisition was performed using custom software written in C++ for rapid frame grabbing, processing and display of two-dimensional images^28,29^. Manual segmentations were used to measure the thicknesses of the mice retinas. During retinal scanning, three volumes (~5 s/vol) were acquired in different sectors of the retina using the ON head as landmark. After processing, three B-scans were randomly selected from each volume.

The retinal thickness measurements were performed with ImageJ software using the saved data. In each B-scan, the thickness of the Nerve fiber layer–Ganglion cell layer (GCL)–Inner plexiform layer, hereafter referred to as NGI, and ONL, where the cell body of the photoreceptors reside, was measured at three adjacent points, as previously described^12^. Measurements of the NGI controlled for the photoreceptor specificity of the damage. Data are shown for WT, RHOP and RHOP:T1 as average ONL thickness in µm ± SEM (absolute values), or as average of percent ONL thickness ± SEM (relative values). For relative values, each vehicle-treated eye (left eye) was set as 100% versus the drug-treated eye (right eye).

### Fluorescence “in situ” hybridization (FISH)

The RNA probes for the “in situ” hybridization were prepared as described. For TrkC.T1, the mouse TrkCT1-specific sequence of exons 13b and 14b and the subsequent 286 bases of the 3′ untranslated region^26^; for NT-3, a 392 basepair region of rat genomic DNA corresponding to bases 481–873 of the rat NT-3 cDNA sequence^30^ was cloned into the pBluescript II KS(+) phagemids; for TNF-α,_._ a 235 basepair region of mouse genomic TNF-α corresponding to bases 837–1072 was synthesized by PCR using Forward: 5‘-gagtccgggcaggtctacttt-3′ and Reverse: 5‘ taatacgactcactatagggagacaggtcactgtcccagcatct-3′ primers for the sense probe; and Forward 5‘- taatacgactcactatagggagagagtccgggcaggtctacttt -3′ and Reverse 5‘-caggtcactgtcccagcatct3′ primers for the antisense probe. Fluorescence “in situ” hybridization was done as previously described^12^. Briefly, retinal sections (20 μm thick) were mounted onto gelatin-coated glass slides, re-fixed in 4% paraformaldehyde (PFA), permeabilized with proteinase K and acetylated. Hybridization was carried by incubating the slides with either 200 ng/ml of digoxigenin-labeled TNF-α or TrkC.T1 or NT-3 antisense RNAs probes overnight at 72 °C in a hybridization oven (Robbins Scientific). As controls, hybridization with either 200 ng/ml of digoxigenin-labeled TNF-α or TrkC.T1 or NT-3 sense RNAs probes was performed on sequential slides in parallel using the same experimental conditions. Images were obtained using an IX81 confocal microscope (Olympus) equipped with Fluoview 281 3.1 software (Olympus).

### Histology, image acquisition and data analysis

The eyes were enucleated and immersed for 1 h in fixative composed of 2% glutaraldehyde in phosphate-buffered saline (PBS) at room temperature. Afterward, cornea was removed and the eye cups were placed in fresh 2% glutaraldehyde and incubated on nutator, overnight at room temperature. The eye cups were dehydrated in ascending grades of reagent alcohol and embedded into epon resin. Sections (800 nm thick) were cut on a LKB ultratome (LKB 2088 Ultrotome V, Sweden) and placed onto Superfrost Plus Slides (Fisher Scientific, ON, Canada). Slides were stained with 1% toluidine blue and mounted with Permount (Fisher Scientific).

Images were collected using the Leica DM LB 2 microscope equipped with the LAS acquisition software and a Leica DFC480 camera for detection, applying a 40× objective. Images were saved directly in TIF format and adjusted using Adobe Photoshop CS 8.0 for unbiased brightness and contrast. For each experimental condition, 6 images were acquired from 3 sections cut from different areas of the retina (*n* = 2 retinas per group) starting at 500 μm from the optic nerve head to avoid thickness variability due to the thinning of the retinal layers close to the boundaries of the optic nerve. The number of photoreceptors nuclei contained inside a rectangle of a fixed area (0.0156 mm^2^), drawn in the ONL, was counted using ImageJ software (developed by Wayne Rasband, National Institutes of Health, Bethesda, MD, USA; http://imagej.nih.gov/ij). Data are shown as the average number of cells per mm^2^ ± SEM.

### Immunohistochemistry, image acquisition and data analysis

After enucleation, the eyes were immersed overnight in fixative composed of 4% PFA in PBS at 4 °C, followed by cryoprotection by soaking in 30% sucrose overnight at 4 °C. Eyes were frozen in optimum cutting temperature (O.C.T.) Tissue TEK and cryostat sections were cut and mounted onto gelatin-coated glass slides. Sections (20 μm thick) were washed with PBS (pH 7.4) and then incubated in PBS containing 5% normal goat serum and/or 5% normal donkey serum, 0.3% Triton X-100 and 0.5% bovine serum albumin (BSA) for 2 h. Afterwards, sections were incubated overnight at 4 °C with primary antibody (Table 1). The 2B7 (anti-TrkC-FL) antibody is specific for the full-length TrkC receptor^23^, whereas TrkC.T1 antibody (Rockland) specifically recognizes the TrkC.T1 isoform, but not full-length TrkC. The sections were rinsed and incubated with secondary antibody (Table 1) for 1–2 h at room temperature. Finally, sections were washed and cover-slipped using Permafluor (Thermo Fisher Scientific, Fremont, CA, USA) or Vectashield mounting media with DAPI.

**Table 1:** Details of antibodies used for immunohistochemistry

Specificity	Source	Clone	Company	Dilution
Primary antibodes				
Protein kinase C	Mouse	MC5	Sigma	1:100
CRALBP	Mouse	B2	Cedarlane	1:200
Glutamine synthetase	Mouse	Monoclonal	Millipore	1:500
p44/p42 MAPK (Erk1/2)	Rabbit	Polyclonal	Cell Signaling	1:25
Phospho p44/p42 MAPK (Erk1/2) (Thr202/Tyr204)	Rabbit	Polyclonal	Cell Signaling	1:200
Phospho-Akt (Ser473)	Rabbit	D9E	Cell Signaling	1:100
Akt	Rabbit	Polyclonal	Cell Signaling	1:1000
TrkCT1	Rabbit	Polyclonal	Rockland	1:100
2B7 (anti-TrkC-FL)	Mouse	Monoclonal	Dr. Saragovi	1:1000
Actin	Rabbit	Polyclonal	Sigma	1:1000
NT-3	Rabbit	Polyclonal	Santa Cruz	1:100
Secondary antibodies				
Rabbit IgG	Goat	Alexa Fluor 488	Invitrogen	1:200
Mouse IgG	Goat	Alexa Fluor 594	Invitrogen	1:200
Mouse IgG	Donkey	Rhodamine-Red-X	ImmunoResearch	1:200
Rabbit IgG	Goat	Peroxidase	Sigma	1:5000

Images were collected using a Leica DMI6000 B microscope equipped with the Quorum technologies WaveFX spinning disk confocal microscopy system, the Volocity software and a high dynamic ImagEM EM-CCD camera for detection. Pictures were taken as Z-stacks of confocal optical sections applying a 20× objective. Images were exported directly in TIF format and adjusted using Adobe Photoshop CS 8.0 for unbiased brightness and contrast.

For each experimental condition, a minimum of 6 images were acquired from 3 sections cut from different areas of the retina (*n* = 3-4 retinas per group). The area of the profiles of the cells expressing p-Akt and p-Erk was measured using ImageJ software. For the “in situ” studies, an arbitrary rectangle of 129 × 97 pixels was drawn for each layer of the retina (GCL, IPL, INL and photoreceptor (PhR)) to measure the TrkC.T1-positive staining using ImageJ. Data are shown as the average area (in pixels) ± SEM or as normalized area values ± SEM, with WT being set to 1 (100%).

### Retinal organotypic cultures

Whole eyes were enucleated and whole retinas dissected from WT and RHOP347S mice at PN day 18. Retinas were used for organotypic culture experiments in 24-well plates containing 500 μl of culture medium (Dulbecco's modified Eagle's medium/F12 supplemented with 10 mM NaHCO_3_, 100 μg/ml Transferrin, 100 μM Putrescine, 20 nM Progesterone, 30 nM Na2SeO_3_, 0.05 mg/ml Gentamicin, 2 mM L-glutamine and 1 mM sodium pyruvate). Under sterile conditions, the media were gently removed and replaced with fresh media containing the treatments or controls, and incubated at 37 °C and 5% CO_2_ for 24 h.

### TUNEL staining

Staining was performed using the DeadEnd Fluorometric terminal deoxynucleotidyl transferase dUTP nick end labeling (TUNEL) system (Promega). Vehicle or KB1368-treated RHOP347S retinas in culture (*n* = 6) were first fixed in 4% PFA in PBS and kept at 4 °C overnight. Then, the sections were washed with PBS–0.2% BSA and permeabilized using 2% Triton X in PBS. Retinas were then incubated with 20 μg/ml proteinase K in PBS for 15 min, briefly re-fixed in PFA for 30 min and washed again with PBS–0.2% BSA before being transferred into Eppendorf tubes. Samples were incubated with 50 μl of equilibration buffer for 20 min, then 25 μl of TdT reaction mixture for 2.5 h at 37 °C. The reaction was terminated using 30 min of incubation of 2× SSC solution. The retinas were washed and mounted between two coverslips with the ganglion cell layer facing up using Vectashield with 4',6-diamidino-2-phenylindole (DAPI) as this was easier to achieve given the natural curvature of the retina. The cover-slipped samples were then flipped over and taped to microscope slides to have the photoreceptor side facing up. For image acquisition, the retinas were divided into 4 quadrants, and 2 pictures with a ×20 objective were taken in each area (one central and one peripheral) for a total of 8 images of the ONL per retina. Total TUNEL-positive cells were counted in each image semiautomatically (ImageJ) by three independent blinded examiners. WT retinal flat mounts were used as negative controls.

### Western blots

Whole retinas either from culture or freshly dissected were collected and added to 200 μl lysis buffer (20 mM Tris-HCl pH 7.5, 137 mM NaCl, 2 mM EDTA, 1% Nonidet P-40) containing a protease inhibitor cocktail (Roche). Samples were sonicated briefly and left on ice for 30 min before centrifuging and obtaining the supernatant. Protein quantities were assessed using the Bradford assay (Bio-Rad), and 40 μg of total protein was mixed with Laemmli buffer, boiled for 5 min and loaded onto 10% gels. After transferring to polyvinylidene difluoride membranes, a blocking step of 1 h in 2% BSA was done, followed by an overnight incubation at 4 °C with the primary antibodies. Membranes were washed repeatedly and then incubated for 1 h with secondary antibody, washed again and developed using Western Lightning Plus ECL (PerkinElmer). Blots were scanned and quantified using ImageJ software. All primary antibodies were used in a 1:2000 dilution and secondary antibodies in a 1:10,000 dilution. To detect total protein for loading controls, membranes were stripped with 62.5 mM Tris-HCl, pH 6.8, 2% sodium dodecyl sulfate and 0.7% 2-Mercaptoethanol at 55 °C for 15 min, followed by extensive washing, blocking, and re-incubation with the primaries as just described.

### Statistical analysis

Results are presented as mean ± SEM for all studies. One-way analysis of variance with significance α = 0.05 or higher were used for processing data. Bonferroni post-hoc analysis was used for calculating significance between groups. Two-tailed Student's *t-*test was used to test for significance between two means.

## Results

### Rhodopsin mutation causes progressive thinning of the ONL

**Fig. 1:**
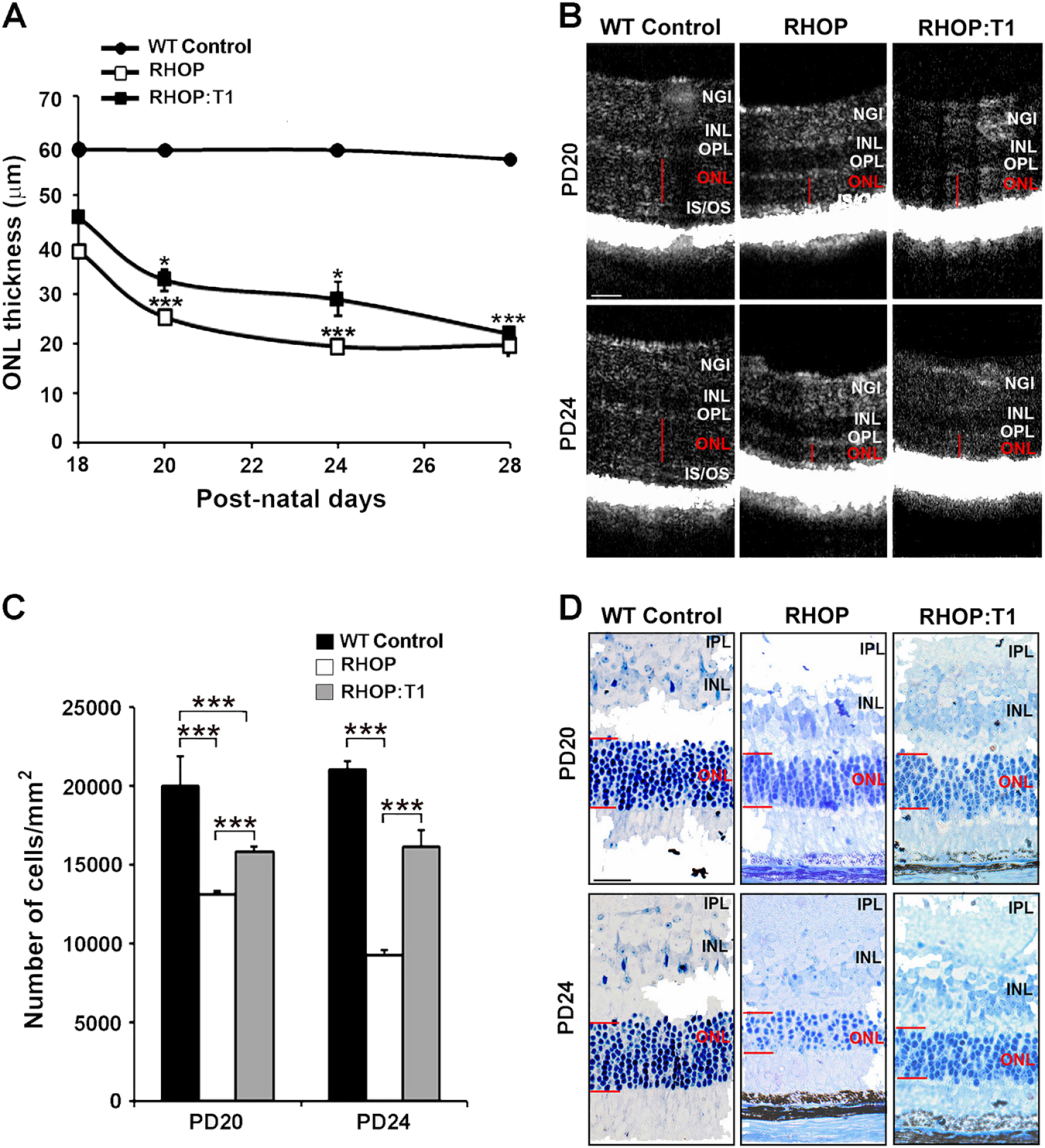
TrkC.T1 depletion delays degeneration of the ONL during RP **a** ONL average thickness measurements ( ± SEM) from FD-OCT images at PN days 18, 20, 24 and 28. The ONL thickness decreases over time in RHOP retinas compared to WT control. Note that the thinning of the retina is delayed in RHOP:T1 retinas compared with RHOP, *n* = 8–10 mice per group, ***p < 0.001 (RHOP versus WT) and *p < 0.05 (RHOP versus RHOP:T1). **b** FD-OCT representative retina images from WT, RHOP and RHOP:T1 mice at PN days 20 and 24, scale bar = 30 µm. **c** Quantification of the number of photoreceptors per mm^2^ (average ± SEM) in ultrathin retinal sections. A total of 18 images were taken from *n* = 2 retinas per group, ***p < 0.001. Images were taken at ×40. **d** Representative images of ultrathin retinal sections at PN days 20 and 24, scale bar = 60 µm. Note that photoreceptor cells are stained in blue. NGI Nerve fiber layer–Ganglion cell layer–Inner plexiform layer; IPL inner plexiform layer, INL inner nuclear layer, ONL outer nuclear layer, IS/OS internal/external segment

The thinning of the ONL is detectable as early as PN day 18, and rapidly decreases by ~33% (*p* < 0.001) at PN day 24, and by ~60% (*p *< 0.001) at PN day 28, compared to WT retinas (Fig. 1a). NGI measurements were 46.13 μm ± 0.727 in WT mice, and 46.71 μm ± 0.98 in RHOP mice at PN day 24. Therefore, these retinal layers are not affected in RP. These data corroborate that rhodopsin mutation causes progressive thinning exclusively in the ONL, but not in rhodopsin-unrelated retinal layers such as the NGI. The PN day 28 was the experimental end point because the ONL is no longer measurable unbiasedly beyond this time point.

### TrkC.T1 heterozygosity delays thinning of the ONL in RHOP mice

We previously demonstrated that deletion of TrkC.T1 reduces RGC death in glaucoma, a model where RGCs are stressed and degenerate^22^. Thus, we examined whether TrkC.T1 has an impact on the degeneration of the ONL in RP.

FD-OCT data showed that there is a significant delay in ONL thinning in RHOP:T1 mice compared to their RHOP littermates, from PN day 18 to PN day 24 (Fig. 1a, b). However, at PN day 28, the ONL thickness was similar in both RHOP and RHOP:T1 retinas (Fig. 1a). Similar results were obtained using quantitative histochemical techniques. (Fig. 1c, d).

These data indicate that the deletion of TrkC.T1 has a temporary protective role in RP, and that 50% reduction of TrkC.T1 is sufficient to delay degeneration of the ONL in RHOP:T1 retinas.

### TrkC.T1 is upregulated in retina glia Müller cells of RHOP mice

**Fig. 2:**
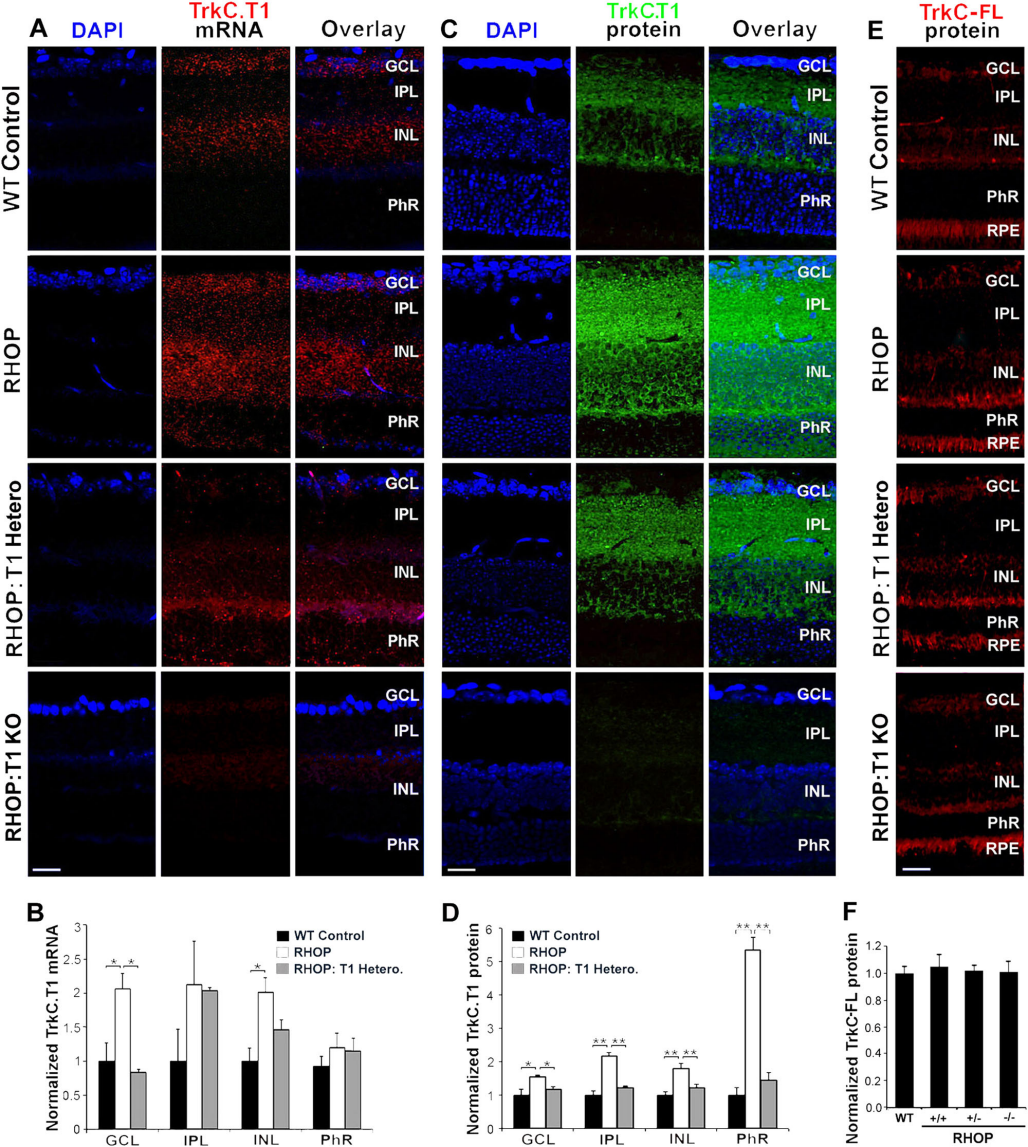
TrkC.T1 mRNA location and protein expression in healthy and RHOP retinas **a** Fluorescence “in situ” hybridization (FISH) with TrkC.T1 mRNA antisense in WT, RHOP, RHOP:T1 heterozygote and RHOP:T1 KO (full TrkC.T1 knockout) mice at PN day 24. **b** Histogram shows the quantification of the area of TrkC.T1 mRNA signal in the different retina layers. TrkC.T1 was markedly increased in the GCL and INL in RHOP retinas and partially decreased in RHOP:T1. Data are represented as normalized TrkC.T1 mRNA area values ( ± SEM) relative to WT control values in each retina layer. A total of 18 images were taken from an *n* = 3–4 retinas per group. **c** TrkC.T1 immunoreactivity in WT, RHOP, RHOP:T1 heterozygote and RHOP:T1 KO (full TrkC.T1 knockout) mice at PN day 24. **d** Quantification of TrkC.T1 immunoreactivity in the different retina layers. TrkC.T1 immunoreactivity was increased in the GCL, IPL, INL and PhR of RHOP mice retinas, whereas TrkC.T1 was decreased in RHOP:T1 eyes. Data are shown as normalized TrkC.T1 protein area values ( ± SEM) relative to WT control in the different retina layers. A total of 30–40 images were taken from an *n* = 3–4 retinas per group. **e** TrkC-FL immunoreactivity in WT, RHOP, RHOP:T1 heterozygote and RHOP:T1 KO (full TrkC.T1 knockout) mice. **f** Histogram shows the quantification of the area of TrkC-FL protein in the retina. No changes were observed among the different groups. Data are shown as normalized TrkC-FL protein area values ( ± SEM) relative to WT control. RHOP+/+: RHOP, wild-type TrkC.T1; RHOP+/−: RHOP, TrkC.T1 heterozygote; RHOP −/− RHOP, full TrkC.T1 knockout. A total of 10 images were taken from *n* = 3 retinas per group. *P < 0.01 and **p < 0.001. Images taken at ×20. Scale bar = 25 µm. GCL ganglion cell layer, IPL inner plexiform layer, INL inner nuclear layer, ONL outer nuclear layer, PhR photoreceptor layer

We have shown that TrkC.T1 is upregulated in glial cells in glaucoma, and in mouse and human ALS^22,23^. Hence, we studied TrkC.T1 localization of mRNA and protein in WT and RP mice. In WT retinas there are low levels of TrkC.T1 mRNA in the GCL and INL. In RHOP retinas, there was an increase of TrkC.T1 mRNA (*p* ≤ 0.01) in the GCL and INL (Fig. 2a quantified in Fig. 2b). In RHOP:T1 retinas there was a significant reduction of TrkC.T1 mRNA by ~50% in the GCL (*p* ≤ 0.01) compared to RHOP mice, and a reduction of TrkC.T1 mRNA by ∼25% in the INL (not statistically significant). There were no reductions in the IPL and the PhR layer, suggesting that there may be a preferential accumulation of TrkC.T1 mRNA in these anatomical sites. Control hybridization with TrkCT1 antisense mRNA in RHOP retinas fully depleted of TrkC.T1 (RHOP:T1 KO) and hybridization with sense TrkC.T1 mRNA in WT and RHOP retinas were negative (data not shown).

RHOP retinas had a robust increase of TrkC.T1 immunoreactivity in the IPL, and a moderate increase in the GCL, INL and PhR layers, compared with WT retinas. Interestingly, TrkC.T1 staining was detected surrounding the cell somata in the GCL, and intermingled with photoreceptors in the PhR layer. In RHOP:T1 mice, TrkC.T1 immunoreactivity was partially reduced in the GCL, IPL, INL and PhR layerS (*p* < 0.01), compared to RHOP retinas (Fig. 2c quantified in Fig. 2b). TrkC.T1 signal was completely absent in RHOP retinas with full knockout TrkC.T1, confirming the specificity of the TrkC.T1 antibody.

There were no significant differences in TrkC-FL protein between WT retinas and any of the RHOP genotypes (Fig. 2e quantified in Fig. 2f). TrkC-FL protein was present in neuronal retinal layers.

The TrkC.T1 immunohistochemistry results were consistent with the in situ mRNA hybridization data with the exception that the TrkC.T1 protein was strongly expressed in the IPL and PhR (Fig. 2c, d), whereas TrkC.T1 mRNA levels were low in these areas. This observation suggests that TrkC.T1 protein may be transported from the Müller cell soma to the Müller fibers contacting neurons in the inner and the outer nuclear layers of RHOP mice.

These results demonstrate that TrkC.T1 is upregulated in glia during RP and that this upregulation takes place mainly in the INL and in the projections of Müller cells towards the IPL and PhR layers where photoreceptors reside. This change is TrkC.T1 happens without obvious alterations in TrkC-FL expression.

### The NT-3 is upregulated in RHOP mice

NT-3 binds and activates TrkC-FL and TrkC.T1 with equal affinity^26^. Thus, we analyzed the NT-3 mRNA expression and its distribution.

**Fig. 3:**
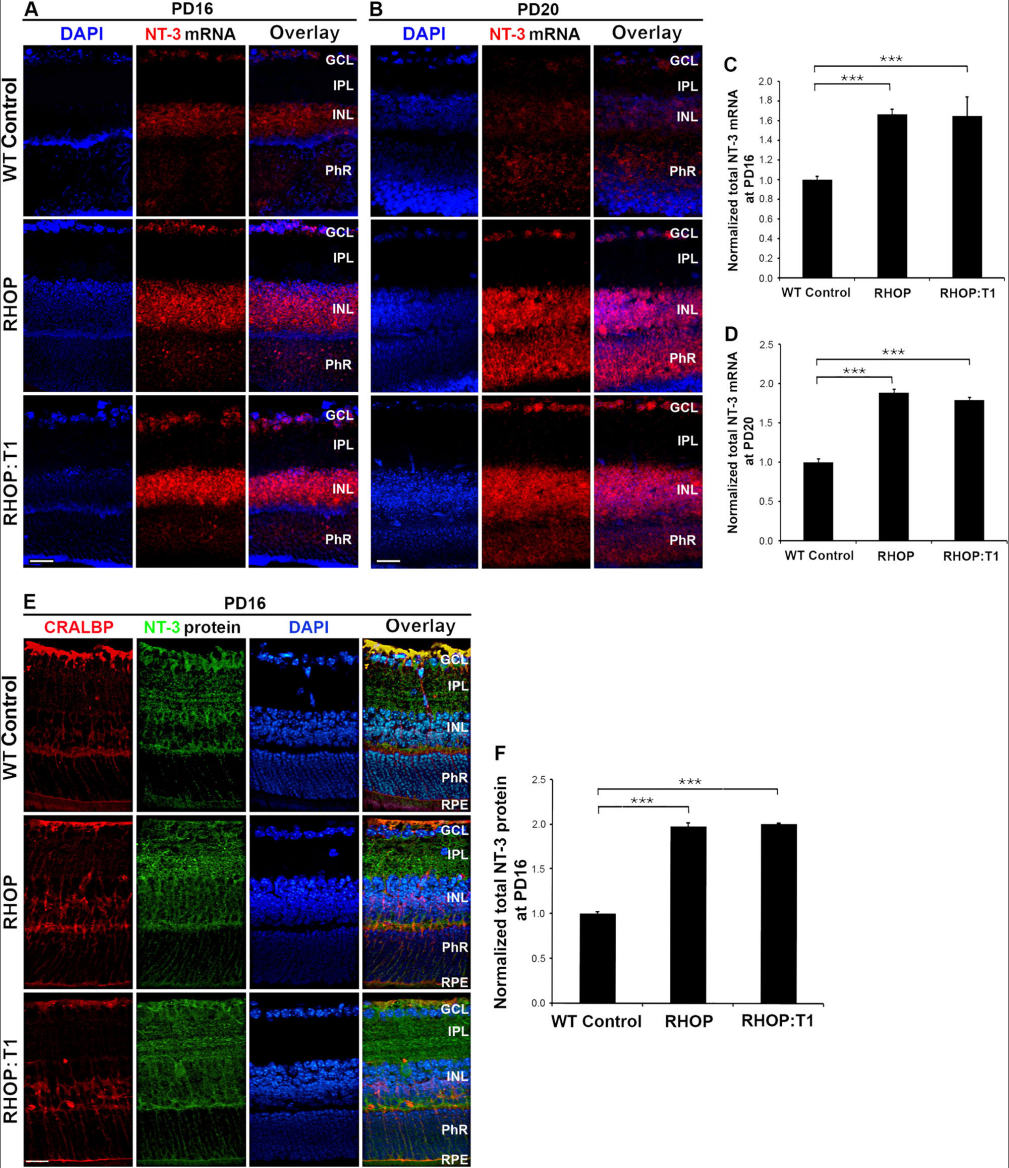
NT-3 is upregulated in RHOP mice **a** Representative image of “in situ” mRNA hybridization with NT-3 (red) antisense probe in WT, RHOP and RHOP: T1 mice at PN day 16 and **b** at PN day 20. NT-3 mRNA was increased in the GCL, INL and more prominently in the PhR layer of both RHOP and RHOP:T1 retinas compared to WT control. **c** Quantification of NT-3 mRNA in total retina at PN day 16 and **d** at PN day 20. Note that the NT-3 mRNA levels in RHOP and RHOP:T1 retinas are not significantly different. Data are shown as normalized area values ( ± SEM) relative to WT control. A total of 16–20 images were taken from an *n* = 2 retinas per group, ***p < 0.001. **e** Representative images of double immunofluorescence with NT-3 (green) and the Müller cell marker CRALBP (red) in WT, RHOP and RHOP:T1 mice at PN day 16. NT-3 immunoreactivity was increased in the IPL, INL and more prominently in the PhR layer of RHOP and RHOP:T1 mice retinas, but not in the GCL. Note that NT-3 immunostaining was detected in the somata of Müller cells, basal end feet and mostly in the fibers projected towards the photoreceptors. Images were taken at ×20. Scale bar = 25 µm. **f** Quantification of NT-3 immunoreactivity in total retina. Data are shown as normalized area values ( ± SEM) relative to WT control. A total of 16-20 images were taken from an *n* = 2 retinas per group, *p < 0.05, **p < 0.01 and ***p < 0.001. GCL ganglion cell layer, IPL inner plexiform layer, INL inner nuclear layer, ONL outer nuclear layer, PhR photoreceptor layer

In WT retinas, we found low levels of NT-3 mRNA in the GCL, INL and PhR layers (Fig. 3a, b). In RHOP and RHOP:T1 retinas at PN day 16 and more noticeably at PN day 20, the levels of NT-3 mRNA were increased to a similar degree and significantly over WT retinas (*p* < 0.001) (Fig. 3c, d). The elevation of NT-3 mRNA levels was detected most prominently in the PhR layer (Fig. 3a, b), and was also detectable in the GCL and INL.

In WT retinas, there was a weak expression of NT-3 protein in the GCL and INL, and undetectable levels in the PhR layer (Fig. 3e). In RHOP retinas (Fig. 3f) and RHOP:T1 retinas (Fig. 3e) there was a significant increase of NT-3 protein in the IPL, INL and even more robustly in the PhR compared to WT retinas (*p* < 0.001). The increased NT-3 signal in RP colocalized with Müller cells, predominantly in Müller cell fibers intermingled with photoreceptors.

These results indicate that NT-3 is specifically elevated early after eye opening in RHOP mice, regardless of the TrkC.T1 genotype, and that the increase takes place mainly towards the damaged photoreceptors, but sparing GCL.

### TrkC.T1 directly activates the MAPK/Erk signaling pathway in Müller glial cells in RHOP mice

Next, we investigated the underlying signaling mechanisms upon TrkC.T1 upregulation in Müller glial cells. TrkC.T1 induces Rac1^26^ and p-Erk and p-Akt are targets of Rac1 in the nervous system^31–33^. Hence, we investigated whether TrkC.T1 regulates p-Erk and p-Akt in the rMC-1 rat Müller cell line, endogenously expressing TrkC.T1 but undetectable levels of TrkC.FL^23^.

**Fig. 4:**
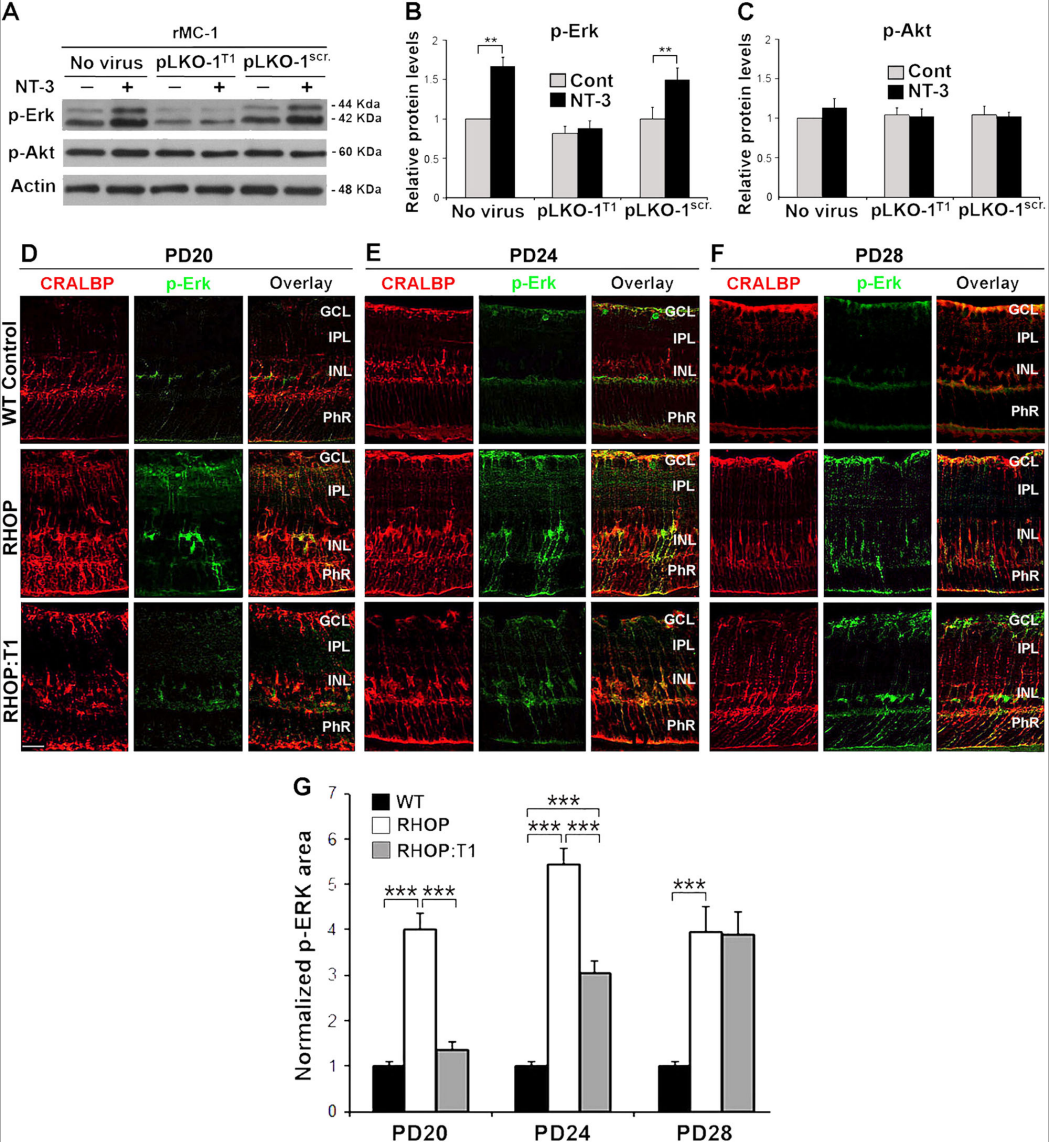
TrkC.T1 directly activates MAPK/Erk in Müller cells **a** Representative western blots showing the levels of TrkC-FL (140 KDa), TrkC.T1 (110 kDa), p-Erk 1/2 (44 and 42 KDa) and p-Akt (60 KDa) in non-infected r-MC1 cells and rMC-1 cells infected with PLKO.1^TrkC.T1^ or lentivirus PLKO.1^scrambled^; with or without stimulation of 4 nM NT-3 for 10 min. All membranes were stripped and re-probed with anti-actin as a loading control. **b**, **c** Densitometric quantification of p-Erk and p-Akt signal standardized to untreated controls (arbitrary value of 1). Note that in rMC-1 cells, silencing TrkC.T1 prevents p-Erk activation. The data represent relative protein levels ± SEM. *n* = 3 independent experiments. *P < 0.05, **p < 0.01 and ***p < 0.001. **d–f** p-Erk immunoreactivity in WT, RHOP and RHOP:T1 at PN days **d** 20, **e** 24 and **f** 28. The p-Erk signal was markedly increased in Müller cells in RHOP retinas at all PN days, as revealed by colocalization with the specific Müller cell marker CRALBP. Note that p-Erk immunostaining was detected in the somata of Müller cells, basal end feet and in the fibers projected towards the outer retinal layers. The p-Erk immunoreactivity was partially decreased in RHOP:T1 retinas. **g** Quantification of p-Erk area in WT, RHOP and RHOP:T1 at PN days 20, 24 and 28. Data are shown as normalized area values vs WT p-Erk area values ( ± SEM). Area was significantly increased in RHOP retinas at all PN days tested. Note that RHOP:T1 retinas showed a significant reduction of p-Erk area at PN days 20 and 24. No difference in area values were observed between RHOP and RHOP:T1 at PN day 28. Thirty images taken from *n* = 3 retinas per group, ***p < 0.001. Images taken at ×20. Scale bar = 25 µm. GCL ganglion cell layer, IPL inner plexiform layer, INL inner nuclear layer, ONL outer nuclear layer, PhR photoreceptor layer

The rMC-1 cells were untreated or infected with either PLKO.1^TrkC.T1^ or control PLKO.1^scrambled^, and each culture was stimulated with vehicle or 4 nM NT-3 for 10 min. In uninfected cells and control lentivirus PLKO.1^scrambled^ infected cells NT-3 stimulation induced an ∼2-fold increase and an ∼1.8-fold increase in p-Erk, respectively. However, there was no NT-3-stimulated increase in p-Erk in cells infected with PLKO.1^TrkC.T1^ (Fig. 4a, b). As control, the levels of p-Akt did not change under any culture condition (Fig. 4a, c). Overall, these results support the view that the MAPK/Erk signaling cascade is activated in a ligand-dependent manner by TrkC.T1 in Müller glial cells.

Next, we investigated whether TrkC.T1 regulates p-Erk in vivo early during RP. In healthy WT retinas, a very weak p-Erk signal was detected in the INL and GCL. In RHOP retinas, there was a sustained increase of p-Erk clearly colocalized with Müller glial (Fig. 4d–f). In the early RHOP retinas, at PN day 20, the p-Erk signal was found predominantly in the soma of Müller glial cells, and in Müller cell fibers and Müller cell end feet in the direction of and spanning the PhR layer (Fig. 4d). In the RHOP retinas at PN day 24, relatively late in disease, p-Erk was even more robust in Müller cell bodies and fibers projected towards the PhRs and in their processes ending in basal end feet (Fig. 4e). In the RHOP retinas at PN day 28, the p-Erk immunoreactivity was decreased (Fig. 4f).

In RHOP:T1 retinas at PN days 20 and 24, there was a reduction of p-Erk signal in the somata and Müller cell fibers compared with RHOP retinas (*p *< 0.01). However, the p-Erk levels were still higher in RHOP:T1 than in WT. In RHOP:T1 retinas at PN days 28, there were no significant differences in p-Erk compared to RHOP retinas (Fig. 4g). These genetic and anatomical data demonstrate that during RP progression, upregulated TrkC.T1 stimulates p-Erk activity specifically in Müller cell fibers pointing towards stressed photoreceptors.

### TrkC.T1 regulates the production of TNF-α mRNA in RHOP mice

Next, we sought to examine whether TrkC.T1 regulates TNF-α mRNA production in RHOP glia. In WT retinas, a weak expression of TNF-α and TrkC.T1 was detected in the GCL and INL. At PN day 16, in RHOP retinas, there were increased levels of TNF-α mRNA and TrkC.T1 mRNA in the GCL, and more prominently in the INL and PhR layers. TNF-α and TrkC.T1 mRNAs were almost completely colocalized.

**Fig. 5:**
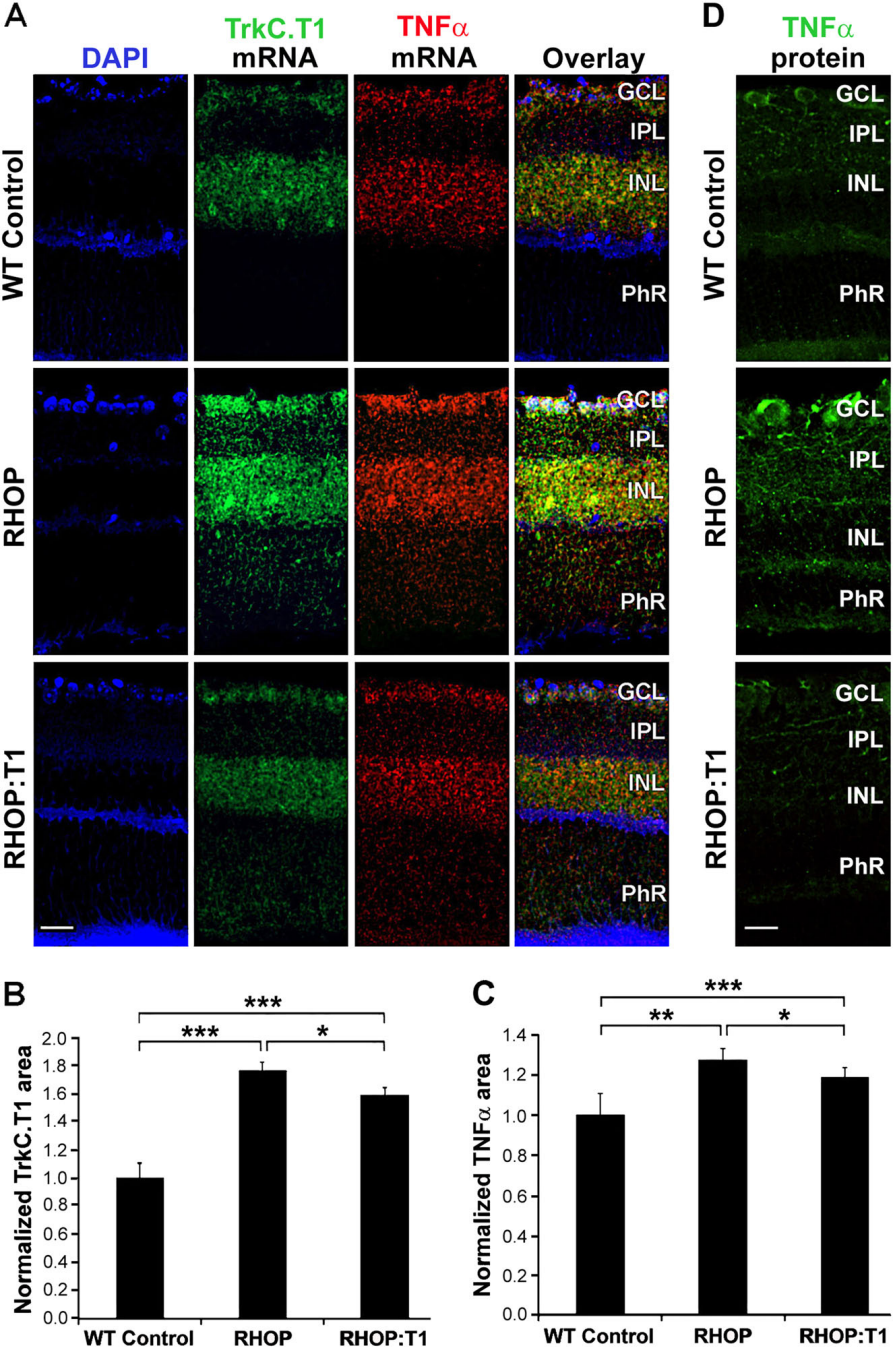
TrkC.T1 mediates TNF-α mRNA upregulation in RHOP mice **a** Double “in situ” mRNA hybridization with TrkC.T1 (green) and TNF-α (red) antisense probes in WT, RHOP and RHOP: T1 mice at PN day 16. TNF-α mRNA was upregulated in the GCL, INL and PhR layer in RHOP retinas. TNF-α upregulation shared the same anatomical distribution of TrkC.T1 mRNA expression, as shown by substantial colocalization between both probes. Note that TNF-α mRNA expression was partially reduced in RHOP:T1 mice. **b** Quantification of TrkC.T1 and **c** TNF- α mRNAs at PD16. Data are represented as normalized TrkC.T1 or TNF-α area values ( ± SEM) relative to WT control. A total of 10 images were taken from an *n* = 3 retinas per group, *p < 0.05; **p < 0.01; ***p < 0.001. **d** TNF-α immunoreactivity in WT, RHOP and RHOP:T1 at PN day 24. Images were taken at ×20. Scale bar = 25 μm. GCL ganglion cell layer, IPL inner plexiform layer, INL inner nuclear layer, PhR photoreceptor layer

In RHOP:T1 retinas, there was a reduction of TrkC.T1 mRNA accompanied by a reduction in TNF-α mRNA, mostly in the INL (Fig. 5a, quantified in 5b, c). Negative controls by double in situ hybridization with sense mRNAs of TrkC.T1 and TNF-α had no signal, as expected (data not shown). These results were corroborated by analysis of TNF-α protein by immunofluorescence. TNF-α protein was increased in RHOP sections and that increase was thwarted in T1 heterozygotes at all time points tested (Fig. 5d and data not shown). TNF-α receptors are highly expressed in ganglion cells, and are also expressed in photoreceptors. This explains why the staining for TNF-α protein is more apparent in the GCL than in the PhR layer, since soluble TNF-α binds to the receptors in neurons.

These data indicate that there is an anatomical overlap for TrkC.T1 and TNF-α mRNAs in Müller glial cells. Upregulation of TrkC.T1 drives TNF-α mRNA and protein overexpression at early stages of disease in RHOP mice. Partial genetic depletion of TrkC.T1 impedes TNF-α mRNA and protein overexpression. High levels of TNF-α are well known to trigger neuronal cell death in the retina^6,22,34,35^, and hence we evaluated whether TNF-α is upregulated in Müller cells downstream of TrkC.T1 and p-Erk activity.

### In RP, pharmacological antagonism of TrkC prevents p-ERK activation and TNF-α elevation and delays retinal degeneration

We tested the hypothesis that pharmacological antagonism of TrkC may ablate the degenerative phenotype in RHOP retinas, leading to neuroprotection.

**Fig. 6:**
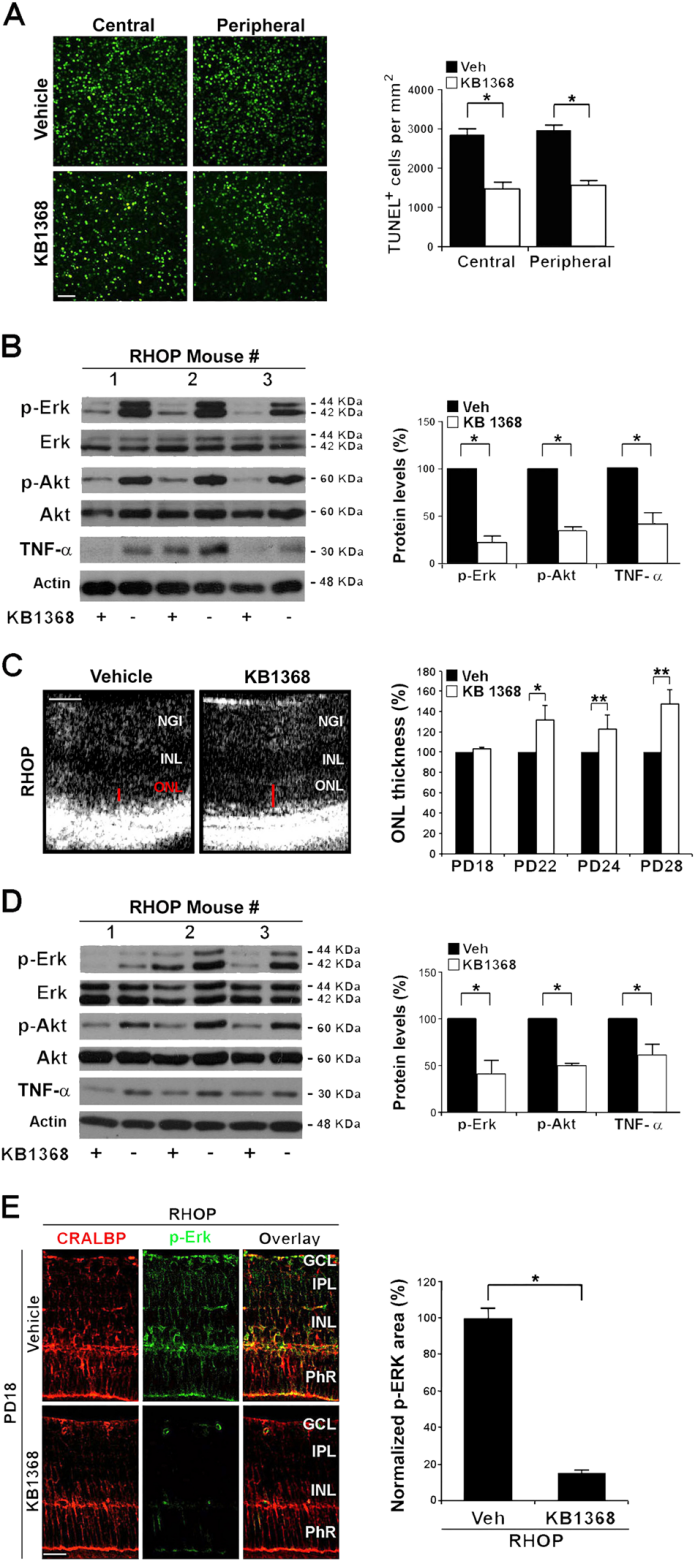
TrkC antagonist KB1368 blocks the expression of p-Erk and p-Akt, decreases TNF-α production and inhibits ONL degeneration and PhR cell death in RP **a**,** b** KB1368 treatment of organotypic retinal cultures. Retinas were dissected at PN day 17 and cultured for 24 h with KB1368 (10 µM, 2 µl) or vehicle (Veh) (5% DMSO, 2 µl). Explants were processed for TUNEL or western blots. **a** Representative images of TUNEL staining of central and peripheral areas of RHOP mice retinas. Histogram shows TUNEL data quantification of both retina areas, TUNEL-positive cells per mm^2^ ( ± SEM), *p < 0.01, *n* = 6. KB1368-treated retinas had decreased photoreceptor apoptosis compared to vehicle. **b** Expression of p-Erk and p-Akt protein in RHOP retinas. Histogram shows densitometric analysis of western blots. The signal for each eye was respectively adjusted to total Erk or total Akt, and the ratio of right/left eye was calculated. In each mouse, the KB1368-treated eye was compared to the vehicle-injected eye standardized to 100% ( ± SEM), *p < 0.01, *n* = 3. The TrkC antagonist reduced p-Erk signal by 70% and p-Akt by 40%. **c–e** Effect of KB1368 in vivo. KB1368 (1 mM, 2 µl) (right eye) or vehicle control (Veh) (5% DMSO, 2 µl) (left eye) were injected intravitreally at PN day 17. **c** FD-OCT images (PN day 24) from RHOP mice. In RHOP eyes KB1368 treatment resulted in ONL thicker than in vehicle-treated from postnatal day 22 to PN day 28 (red bars). Histogram shows FD-OCT data quantified for RHOP retinas at PN days 18, 22, 24 and 28. For each mouse the vehicle-injected eye was standardized to 100%, *n* = 3 animals per group, *p < 0.01, scale bar = 60 µm. **d** Western blots of RHOP retinas 24 h after intravitreal injections. Histogram shows densitometric quantification of p-Erk, p-Akt and TNF-α. The signal for each eye was respectively adjusted to total Erk, total Akt or actin, and the ratio of right/left eye was calculated. For each mouse, the KB1368-treated eye was compared to the vehicle-injected eye standardized to 100% ( ± SEM), *p < 0.01, *n* = 4. The TrkC antagonist reduced p-Erk signal by 40%, p-Akt by 50% and TNF-α by 60%. **e** Representative images of p-Erk immunoreactivity in RHOP retinas at PN day 18. KB1368 abolished p-Erk immunostaining compared with the vehicle-injected retinas. Histogram shows quantification of p-Erk area in pixels ( ± SEM), *p < 0.001. Scale bar = 25 µm. NGI Nerve fiber layer–Ganglion cell layer–Inner plexiform layer, IPL inner plexiform layer, INL inner nuclear layer, ONL outer nuclear layer, IS/OS internal/external segment

First, we tested this hypothesis using organotypic retinal explants cultured ex vivo. In organotypic cultures of RP retinas the photoreceptors continue to die at the same rate as in vivo. The retinal explants were treated with KB1368, a small molecule antagonist of TrkC^22,36,37^ or control vehicle and photoreceptor death was quantified in TUNEL assays. RHOP retinal explants treated with KB1368 had significantly reduced photoreceptor death compared to vehicle (*p* < 0.01) (Fig. 6a) demonstrating a protective role for TrkC antagonism. RHOP retinas cultured with KB1368 TrkC antagonist had a significant reduction of p-Erk, p-Akt and TNF-α protein (*p* < 0.01) compared to vehicle (Fig. 6b).

Similar data were obtained in vivo (Fig. 6c–e). In RHOP retinas the ONL of KB1368-injected eyes (at PN day 17) was significantly thicker than the vehicle-injected eyes at PN days 22, 24 and 28. These data demonstrate a significant preservation of the photoreceptor structures. In further controls, injection of vehicle or KB1368 in WT eyes caused no significant changes in ONL structure (data not shown).

Quantification showed a reduction of p-Erk (∼40%, *p* < 0.01), p-Akt (∼50%, *p* < 0.01) and TNF-α (∼60%, *p* < 0.01) in KB1368-treated RHOP eyes compared to vehicle-injected contralateral eyes (Fig. 6d). Immunohistochemistry demonstrated that reduction of p-Erk resulting from KB1368 treatment occurs primarily in Müller cells (Fig. 6e).

These pharmacological data are consistent with the data obtained using RHOP:T1 as a genetic TrkC.T1 ablation strategy. The only phenotypic difference between RHOP:T1 ablation and the pharmacological inhibitor KB1368 is a reduction in p-Akt. The decrease in retinal p-Akt may be due to TrkC-FL inhibition by KB1368, because KB1368 can antagonize both TrkC.T1 and TrkC-FL^37^ and p-Akt is downstream of TrkC-FL.

Together, these results indicate that in RP Müller cells upregulate TrkC.T1 and NT-3 and that genetic ablation or pharmacological inhibition of TrkC.T1 reduces p-Erk and TNF-α that are otherwise increased in Müller cells. Hence, TrkC.T1 appears to be upstream of p-Erk and TNF-α. The next question was whether p-Erk is upstream of TNF-α and induces its expression.

### Inhibition of MAPK/Erk activity prevents TNF-α elevation and delays retinal degeneration in RP retinas in vivo

We examined whether direct interference with the MAPK/Erk signaling cascade could reduce TNF-α and prevent photoreceptor degeneration in the RHOP mouse in vivo. An intravitreal injection of the MAPK/Erk inhibitor PD90859 (right eye, treated) or control vehicle (left eye, control) were done at PN day 17, and p-Erk was examined by immunohistochemistry 24 h later (at PN day 18).

**Fig. 7:**
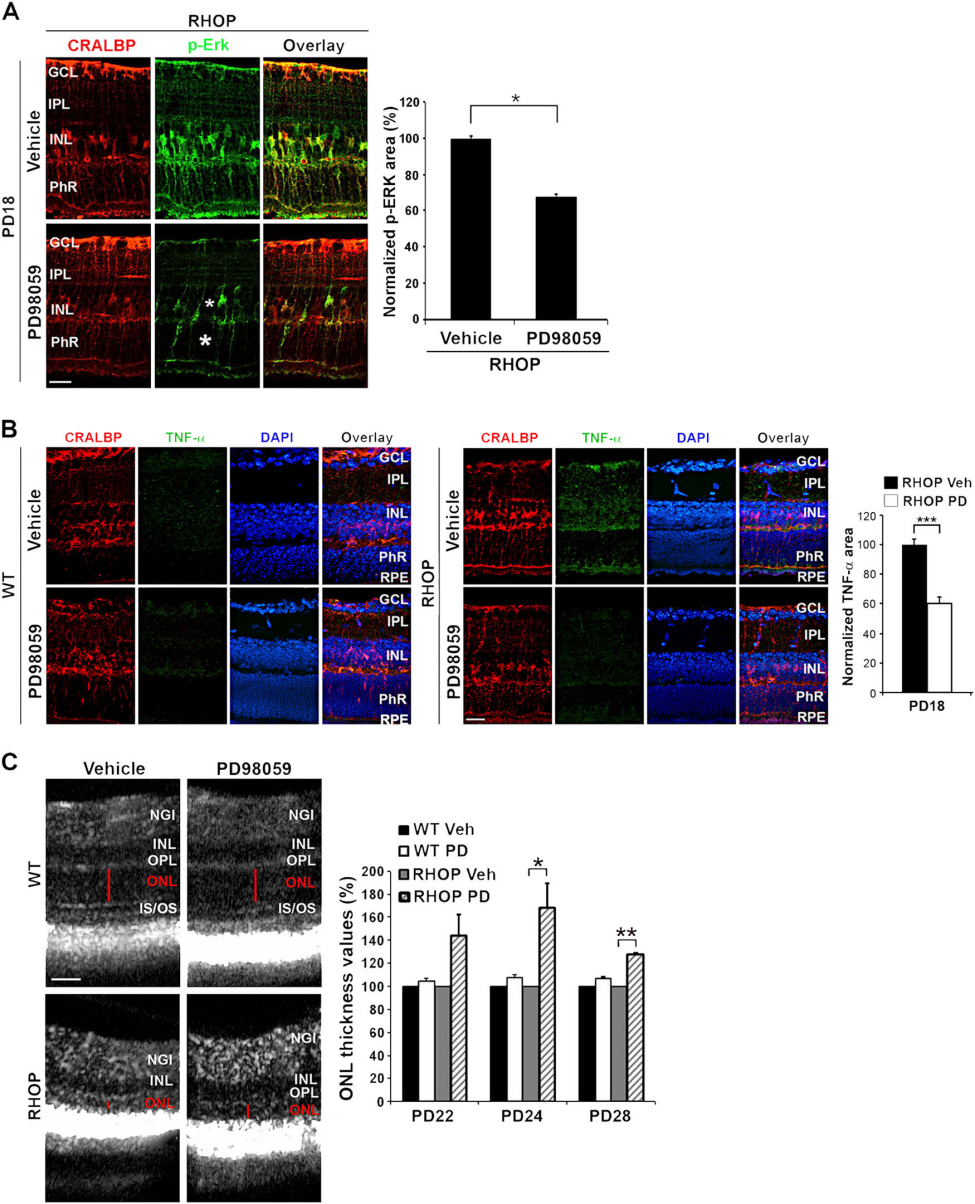
MAPK/Erk inhibition delays ONL degeneration in RP PD98059 (100 µM, 2 µl) (right eye) or vehicle (Veh) (50% DMSO, 2 µl) (left eye, control) were injected at PN day 17. **a** Representative images of p-Erk immunoreactivity in RHOP retinas at PN day 18. P-Erk signal was clearly reduced in the cell bodies and mostly in the fibers of Müller cells projected towards the PhR layer (asterisk) in RHOP eyes treated with PD98059, scale bar = 25 µm. Histogram represents the quantification of the p-Erk area in pixels ( ± SEM), *p < 0.001. **b** Representative images of TNF-α immunoreactivity in RHOP retinas at PN day 18. TNF-α signal was reduced in RHOP eyes treated with PD98059. Histogram shows TNF-α immunofluorescence quantification. Data are shown as normalized TNF-α area values ( ± SEM) vs area values in Vehicle-injected RHOP retinas. **c** FD-OCT representative images (PN day 24) from WT and RHOP mice animals injected with vehicle or PD98059. In PD98059-injected RHOP eyes, ONL was thicker than in vehicle-injected eyes (red bar) at postnatal days 24 and 28. In control WT mice no ONL changes were observed in PD98059-injected compared with vehicle-injected eyes. Histogram shows the quantification of FD-OCT images from WT and RHOP retinas at PN days 22, 24 and 28. In each animal regardless of the genotype, the vehicle-injected eye was standardized to 100%, *n* = 3 animals per group, *p < 0.05 and **p < 0.01, scale bar = 60 µm. NGI Nerve fiber layer–Ganglion cell layer–Inner plexiform layer, IPL inner plexiform layer, INL inner nuclear layer, ONL outer nuclear layer, IS/OS internal/external segment

Treatment significantly blocked p-Erk in Müller cells (*p* < 0.01) compared to control contralateral eye. The p-Erk signal was abolished in Müller cell, most of which contact the photoreceptor layers (Fig. 7a). Inhibition of p-Erk in RHOP mice significantly reduced TNF-α levels, indicating that in the RHOP model TNF-α elevation is p-Erk dependent (Fig. 7b).

Reduction of p-Erk and TNF-α in RHOP mice significantly preserved retinal structure and photoreceptor neurons (Fig. 7c). In PN day 24 RHOP mice the ONL was thicker in PD98059-treated eyes, compared to vehicle-treated contralateral eyes. This indicates significant preservation of retinal structure (*p *< 0.01). ONL protection was still evident at PN day 28 (*p* < 0.001) (note that the higher significance is due to continued degeneration in the vehicle-treated control eyes). In WT control mice the ONL was the same whether eyes were injected with PD98059 or vehicle.

Together, these data demonstrate that in the RHOP model of RP a TrkC.T1-dependent activation of p-Erk in Müller glia, which is vectorial towards photoreceptors, contributes to local TNF-α increases by Müller glia. Elevated local TNF-α causes selective photoreceptor death. Direct inhibition of TrkC.T1 or MAPK/Erk activity prevents local TNF-α increases and delays photoreceptor cell death and degeneration of the ONL.

## Discussion

We report the mechanism of action of TrkC.T1 in a retinal dystrophy mouse model of RP. In disease, TrkC.T1 mRNA and protein are expressed in Müller cells predominantly in an anatomical location proximal to stressed photoreceptors. TrkC.T1 signals are NT-3 dependent, and in disease NT-3 mRNA and protein are expressed predominantly in an anatomical location proximal to stressed photoreceptors. TrkC.T1 is implicated in the sequential activation of MAPK/Erk signaling pathways, with local promotion of TNF-α production in glia. These subsequent events are also vectorial towards stressed photoreceptors, and culminate in their selective death. These data explain a novel mechanism of photoreceptor death in RP, as well as how death signals are selectively delivered to photoreceptors in RP.

It has been challenging to explain how a neuronal population can be affected *selectively* by a paracrine mechanism common to many degenerative diseases, and by a Müller cell that can contact multiple neuronal populations. We provide evidence supporting the “vectorial hypothesis” of Müller cells being activated directionally towards the injured neurons. In addition, we discuss a paired “two hit hypothesis” where Müller cells provide the terminal blow by local delivery of TNF-α which is known to be toxic to an already wounded neuron. In the case of photoreceptors, the first hit is a dystrophic mutant Rhodopsin protein.

### Increased TrkC.T1 in Müller cells in RP

The degeneration of ONL in the retina is a hallmark of RP and reflects the progressive death of photoreceptors^2–4^. In the RHOP model, it takes place during the 2 weeks following eye opening. In the RHOP model the production of NT-3 is increased and TrkC.T1 is upregulated prior to or at the time of eye opening, primarily in Müller cells.

TrkC.T1 and TNF-α mRNAs were increased and were anatomically colocalized in RHOP mice in retinal layers where Müller glial cell bodies reside; at early stages of the disease. These data strongly suggest a correlation between TrkC.T1 and TNF-α upregulation in RP. Here, we also show the molecular mechanism. In Müller cells TrkC.T1 activates p-Erk that causes exacerbated TNF-α production, which causes photoreceptor death and degeneration of the ONL. These events are ameliorated in vivo by genetic reduction of one TrkC.T1 allele, by pharmacological antagonism of TrkC, or by pharmacological inhibition of p-Erk. However, the protective effect of each of these treatments was temporary, most likely due to the complex nature of RP disease, and the convergence of other mechanisms that promote the pathology, such as the neurotoxic activity of p75^NTR^ ^12^.

### Role of p-Erk in Müller cells

In a glial cell line and in RP in vivo p-Erk was regulated by TrkC.T1. The activation of p-Erk in Müller cells has been previously reported in other models of RP^38–40^ and has been identified as a marker of Müller cell proliferation and differentiation^41^, and as an early marker of retinal stress^42–44^. However, there were no data on the mechanism causing p-Erk activation, which we show is TrkC.T1 mediated.

Pharmacological inhibition of the MAPK/Erk cascade reduced TNF-α and delayed photoreceptor death and ONL degeneration in RHOP retinas, indicating that p-Erk activation could ultimately trigger PhR cell death through TNF-α. Our data are consistent with reports showing that specific blockade of TNF-α upregulation reduces photoreceptor cell death in the *rd10* mouse model of RP^6^ and other models of photoreceptor degeneration^45^.

### How do Müller cell neurotoxic signals injure photoreceptors selectively?

Müller cells expand throughout the retina and are layered between the RGCs in the inner retina and the photoreceptors in the outer retina. Given their anatomical position and design, Müller cells sense a healthy state and support neuronal function, or react to neuronal stress or disease ultimately becoming arbiter and executioner^5,23,46^. The selective death of a type of neuron in each disease model (RGCs in glaucoma; photoreceptors in RP), using a single TrkC.T1 mechanism, might be explained by a combination of strategies.

First, Müller cell p-Erk activation is TrkC.T1 dependent and “vectorial” towards the injured neurons. How this may be controlled remains unknown but it could be due to local presence and activation of TrkC.T1, by its ligand NT-3, on the injured side of the retina. Perhaps, there is a gradient of factors or signals stemming from the injured neuron to inform the Müller cell of a stress.

Second, Müller cells may exert a “second hit” on the stressed neuron that tilts the balance towards death. In the case of photoreceptors a “first hit” may include the Rhodopsin mutation that causes dysfunction of light transmission and structural malformations in Rod outer segments. In the case of RGCs in glaucoma a “first hit” may include high intraocular pressure stressing RGC fibers, causing impaired transport and reducing synaptic density. The use of common mechanisms to achieve selective cellular death is not an unusual biological strategy^6^.

### Other models of RP and mechanisms of disease

Our data regarding the role of TNF-α in the RHOP model are consistent with reports of the deleterious effect of glial TNF-α in different RP models^5,6,47,48^. Although RP is primarily a photoreceptor disease, retinal changes beyond photoreceptors have been reported to occur late in disease.

Loss of sensory rod and cone input to the neural retina constitutes deafferentation that results in remodeling at the cellular level and reprogramming at the molecular level and progressive neural degeneration (for review see Jones et al.^46^). Interestingly, bipolar cells undergo anatomical changes that include, among others, the Akt/mammalian target of rapamycin (mTOR) pathway. Akt/mTOR is a key regulator of neuronal structure, function and plasticity^49^ and dysregulation is associated with neurodegenerative states^50^.

### Hypothetical model

**Fig. 8:**
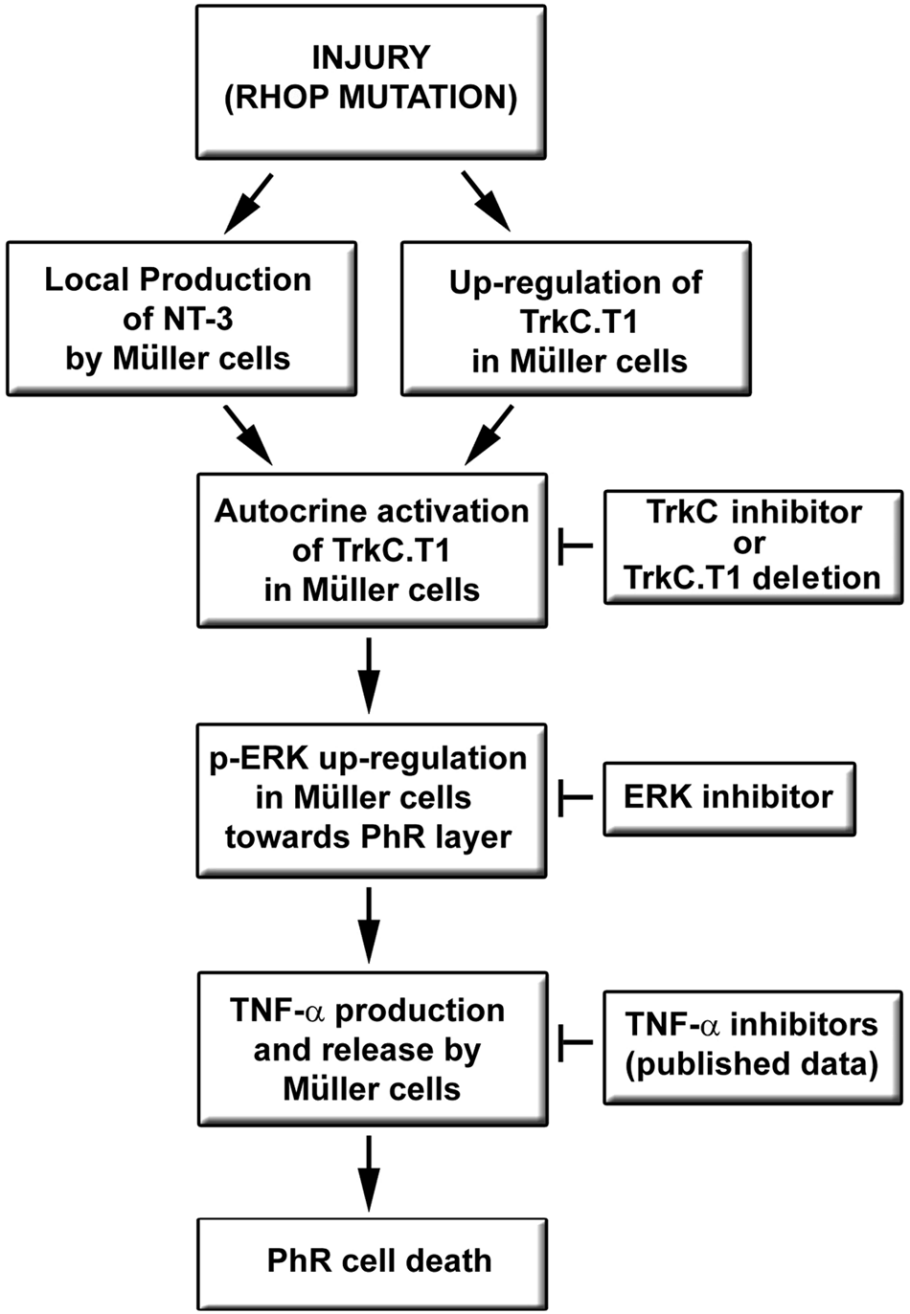
Hypothetical model leading to PhR cell death in RP In RP, photoreceptor stress triggered by the rhodopsin mutation induces the early production of NT-3 and upregulation of TrkC.T1 in Müller glial cells on the injured side of the retina. In Müller glial cells, autocrine activation of TrkC.T1 triggers vectorial expression of p-ERK towards the stressed photoreceptor layer, which in turn promotes the production and release of TNF-α. Genetic ablation of one single copy of TrkC.T1 or intravitreal injections of PD98059 (p-ERK inhibitor) or KB1368 (TrkC antagonist) significantly disminished p-ERK acrivation, decreased TNF-α production and prevented photoreceptor cell death. All these findings represent novel mechanisms. Upregulated TNF-α provokes (and inhibitors of TNF-α delay) photoreceptor cell death (as per literature)

This study shows the implication of TrkC.T1 in one of the most common inherited retinal diseases and uncovers the novel signaling mechanism of TrkC.T1–p-Erk–TNF-α production during disease progression. This represents a non-cell autologous mechanism driven by Müller glia, oriented predominantly towards the injured side of the retina. How glia resolves the *selective death* of a specified neuronal population is paradoxical. Here we postulate a “vectorial hypothesis” and a “two hit hypothesis” (Fig. 8 **)**.

In RP, increased production of NT-3 and local upregulation of TrkC.T1 in Müller cells on the injured side of the retina are the earliest molecular events right after eye opening. In Müller cells, TrkC.T1 activity mediated through NT-3 triggers vectorial expression of p-ERK towards the stressed PhR layer, which in turn promotes the expression/release of TNF-α. Upregulated TNF-α will eventually provoke the cell death of photoreceptors (“vectorial and two hit hypothesis”).

The etiological implication of TrkC.T1 in RP provides a starting point for exploring a shared mechanism in a disease family where more than 200 gene mutations^2–4^ have been identified. Along with our previous evidence in glaucoma, we suggest that TrkC.T1 may be a therapeutic target for the treatment of retinal neurodegenerative diseases.
